# Supplementary material for: Combination of CD49b and CD229 Reveals a Subset of Multipotent Progenitors With Short-Term Activity Within the Hematopoietic Stem Cell Compartment
Source: Stem Cells Transl Med. 2023 Sep 14;12(11):720–6. doi: 10.1093/stcltm/szad057 (PMC10630077; doi:10.1093/stcltm/szad057)
Supplement: szad057_suppl_Supplementary_Material [file szad057_suppl_supplementary_material.pdf]

Figure S1, related to Figure 1-2

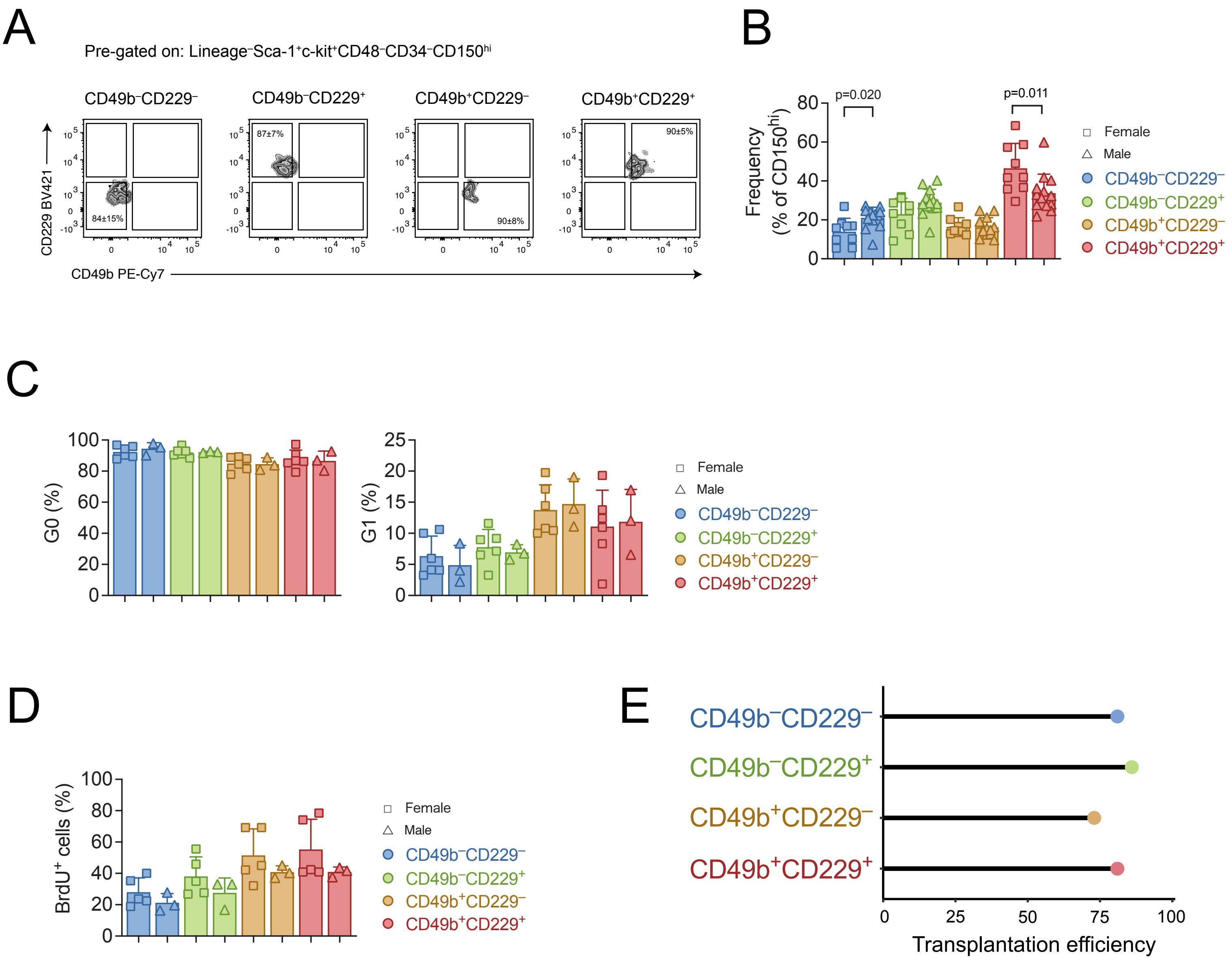

Supplementary Figure S1, related to Figure 1-2. CD49b and CD229 subfractionated subsets in female and male mice. **(A):** Sort purity analysis of $CD49b^{-}CD229^{-}$, $CD49b^{-}CD229^{+}$, $CD49b^{+}CD229^{-}$, and $CD49b^{+}CD229^{+}$ populations pre-gated on $Lineage^{-}Sca\text{-}1^{+}c\text{-}kit^{+}CD48^{-}CD34^{-}$ $CD150^{hi}$ ($CD150^{hi}$). Sort purity is represented as mean ± SD for each subset, from 4 experiments **(B)**: Frequency of $CD49b^{-}CD229^{-}$, $CD49b^{-}CD229^{+}$, $CD49b^{+}CD229^{-}$, and $CD49b^{+}CD229^{+}$ subsets within the phenotypic HSC compartment ($CD150^{hi}$) of young adult mice, separated into females and males. n = 9 females, 13 males from 9 experiments **(C)**: Cell cycle analysis of $CD49b^{-}$ $CD229^{-}$, $CD49b^{-}CD229^{+}$, $CD49b^{+}CD229^{-}$, and $CD49b^{+}CD229^{+}$ subsets by Ki-67 and DAPI staining in young adult mice, separated into females and males. Frequency of cells in G0 (left) and G1 (right) phases are shown. n = 6 females, 3 males from 2 experiments. **(D)**: Cell proliferation analysis by BrdU incorporation in young adult mice, separated into females and males. Frequencies of $BrdU^{+}$ $CD49b^{-}CD229^{-}$, $CD49b^{-}CD229^{+}$, $CD49b^{+}CD229^{-}$, and $CD49b^{+}CD229^{+}$ cells are shown. n = 5 females, 3 males from 2 experiments. **(E)**: Transplantation success rate of transplanted $CD49b^{-}CD229^{-}$, $CD49b^{-}CD229^{+}$, $CD49b^{+}CD229^{-}$, and $CD49b^{+}CD229^{+}$ cells.

Statistical analysis was performed with the Mann-Whitney test in (B-D) between female and male groups within the same subset. All data are represented as mean ± SD. Abbreviations: BrdU, Bromodeoxyuridine.

Figure S2, related to Figure 2

A

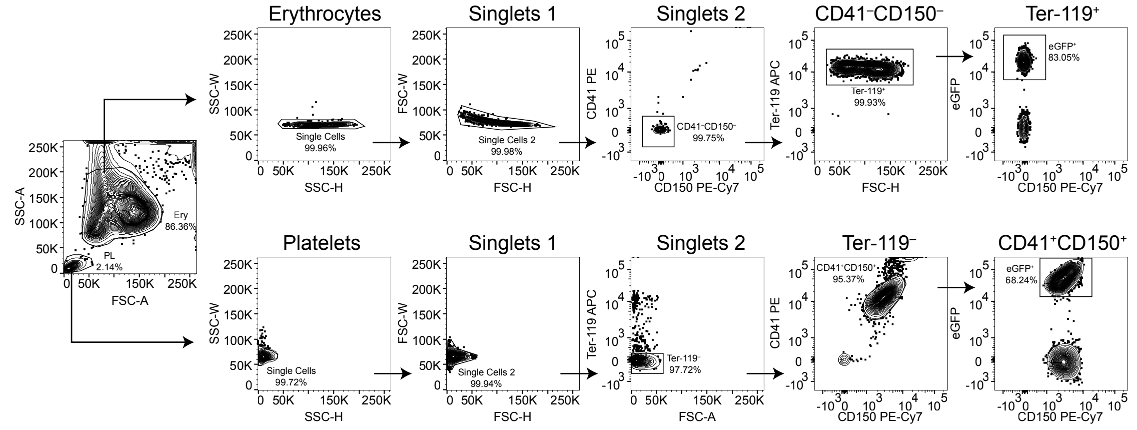

B

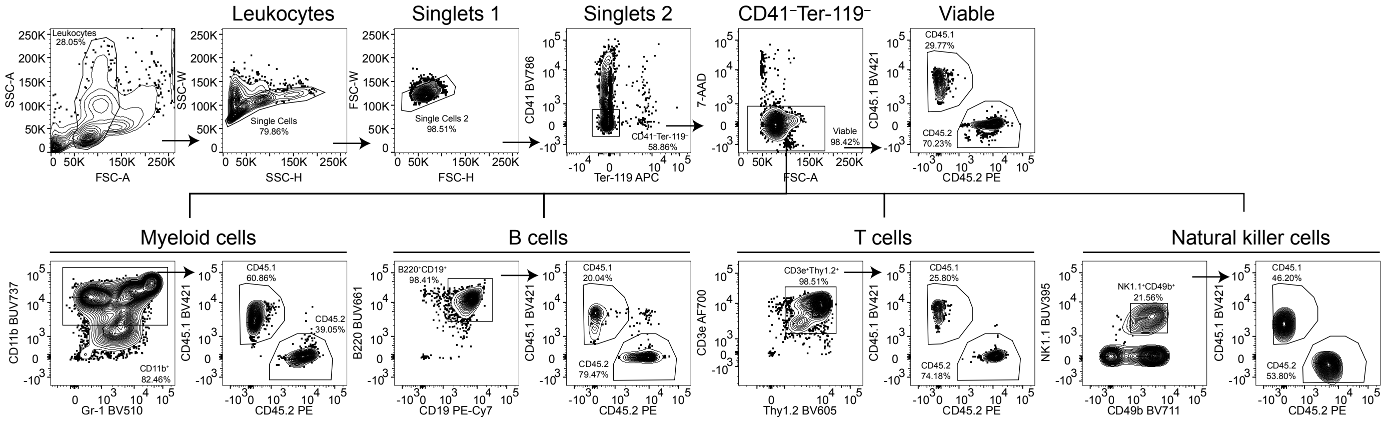

Supplementary Figure S2, related to Figure 2. Flow cytometry analysis and gating strategies. **(A)**: Representative FACS profile and gating strategy of platelet and erythrocyte lineages in the peripheral blood. Platelet: *CD41*$^+$*CD150*$^+$Ter-119$^-$; Erythrocyte: *Ter-119*$^+$CD41$^-$CD150$^-$. **(B)**: Representative FACS profile and gating strategy of myeloid, B, T, and natural killer (NK) cell lineages in the peripheral blood. Donor leukocytes: CD45.2$^+$; myeloid cell: *CD11b*$^+$B220$^-$CD19$^-$NK1.1$^-$CD49b$^-$CD3e$^-$Thy1.2$^-$CD41$^-$Ter-119$^-$; B cell: *B220*$^+$*CD19*$^+$ CD11b$^-$Gr-1$^-$NK1.1$^-$CD49b$^-$CD3e$^-$Thy1.2$^-$CD41$^-$Ter-119$^-$; T cell: *CD3e*$^+$*Thy1.2*$^+$CD11b$^-$Gr-1$^-$NK1.1$^-$CD49b$^-$ B220$^-$CD19$^-$CD41$^-$Ter-119$^-$; NK cell: *NK1.1*$^+$*CD49b*$^+$CD3e$^-$Thy1.2$^-$CD11b$^-$Gr-1$^-$B220$^-$CD19$^-$CD41$^-$Ter-119$^-$.
Frequency of parent gates are shown in all FACS plots.

Figure S3, related to Figure 2

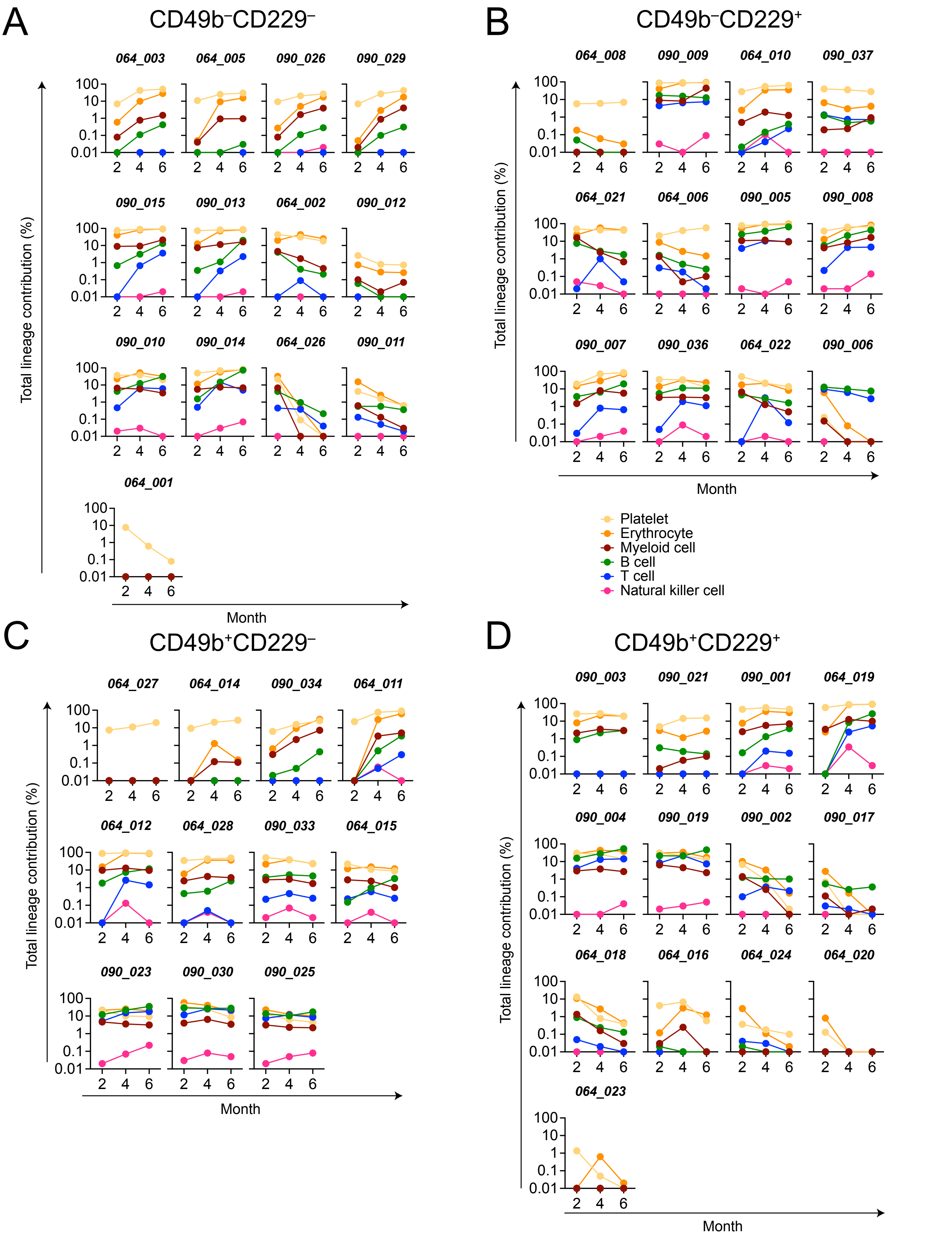

Supplementary Figure S3, related to Figure 2. Blood repopulation patterns of primary transplanted mice. **(A-D)**: Frequency of mature lineage repopulation in peripheral blood 2-, 4-, and 6-months post-transplantation. Individual mice, each transplanted with 5 cells from the $CD49b^{-}CD229^{-}$ subset in (A), $CD49b^{-}CD229^{+}$ subset in (B), $CD49b^{+}CD229^{-}$ subset in (C), and $CD49b^{+}CD229^{+}$ subset in (D) are shown. Data are represented as mean ± SD.

Figure S4, related to Figure 2

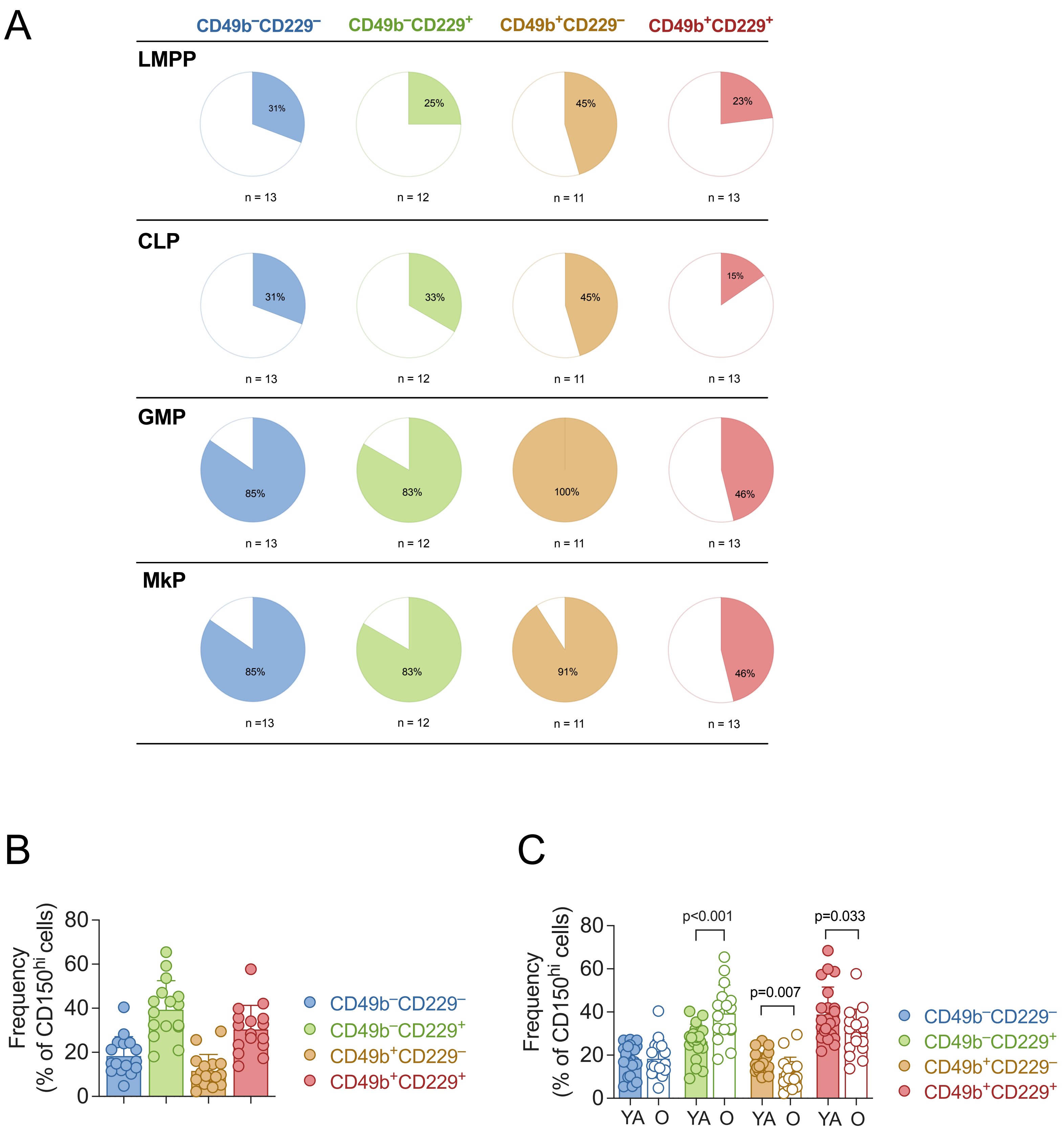

Supplementary Figure S4, related to Figure 2. Repopulation of the bone marrow progenitor compartment and CD49b and CD229 subfractionation in aging. **(A)**: Proportion of mice with LMPP, CLP, GMP, and MkP repopulation 6 months post-transplantation. $n_{CD49b^{-}CD229^{-}}$ = 13 mice, $n_{CD49b^{-}CD229^{+}}$ = 12 mice, $n_{CD49b^{+}CD229^{-}}$ = 11 mice, and $n_{CD49b^{+}CD229^{+}}$ = 13 mice, 2 experiments. **(B)**: Frequency of CD49b^{-}CD229^{-}, CD49b^{-}CD229^{+}, CD49b^{+}CD229^{-}, and CD49b^{+}CD229^{+} subsets within the phenotypic HSC compartment (Lineage^{-}Sca-1^{+}c-kit^{+}CD48^{-}CD34^{-}CD150^{hi}) of old mice. n = 16 biological replicates, 10 experiments. **(C)**: Comparison of the frequency of CD49b^{-}CD229^{-}, CD49b^{-}CD229^{+}, CD49b^{+}CD229^{-}, and CD49b^{+}CD229^{+} subsets in young adult and old mice from Fig. 1B and Fig. S4B.

Statistical analysis was performed with the Mann-Whitney test in (C). Data are represented as mean ± SD in (B-C). Abbreviations: LMPP (Lymphoid-primed multipotent progenitor): Lineage^{-}Sca-1^{+}c-kit^{+}Flt-3^{hi}; CLP (Common lymphoid progenitor): Lineage^{-}B220^{low}Sca-1^{low}c-kit^{low}Flt-3^{hi}IL-7Ra^{+}; MkP (Megakaryocyte progenitor): Lineage^{-}Sca-1^{-}c-kit^{+} (LK) CD150^{+}CD41^{+}; GMP (Granulocyte-monocyte progenitor): LK CD41^{-}CD150^{-}CD16/32^{+}; YA, young adult; O, old.

Supplementary Table S1. Antibody list

Antigen	Clone	Supplier	Order number
**Donor and recipient**			
Anti-mouse CD45.2 (Ly5.2) PE	104	BioLegend	109808
Anti-mouse CD45.1 (Ly5.1) BV421	A20	BD Biosciences	563983
Anti-mouse CD45.1 (Ly5.1) BUV395	A20	BD Biosciences	565212
**Live/Dead, cell proliferation and cell cycle**			
7-AAD		BD Biosciences	559925
DAPI		ThermoFisher Scientific	D3571
BrdU PE		BD Biosciences	556029
Anti-mouse/Human Ki-67 PE		BD Biosciences	567719
Ki-67 FITC		BD Biosciences	556026
**Hematopoietic cells**			
Anti-mouse CD19 PE-Cy7	6D5	BioLegend	115520
Anti-mouse CD45R/B220 BUV395	RA3-6B2	BD Biosciences	563793
Anti-mouse CD45R/B220 BUV661	RA3-6B2	BD Biosciences	565077
Anti-mouse/Human CD45R/B220 PE-Dazzle 594	RA3-6B2	BioLegend	103258
Anti-mouse/Human CD45R/B220 BV510	RA3-6B2	BioLegend	103248
Anti-mouse CD11b (Mac-1) BUV395	M1/70	BD Biosciences	563553
Anti-mouse CD11b (Mac-1) BUV737	M1/70	BD Biosciences	564443
Anti-mouse/Human CD11b (Mac-1) BV510	M1/70	BioLegend	101263
Anti-mouse F4/80 APC	BM8	BioLegend	123116
Anti-mouse F4/80 APC	BM8	ThermoFisher Scientific	17-4801-82
Anti-mouse Gr-1 BV510	RB6-8C5	BioLegend	108437
Anti-mouse Gr-1 BUV395	RB6-8C5	BD Biosciences	563849
Anti-mouse Gr-1 (Ly-6G/Ly-6C) PE	RB6-8C5	BD Biosciences	553128
Anti-mouse Thy1.2 BV605	53-2.1	BioLegend	140318
Anti-mouse CD3e AF700	500A2	BD Biosciences	557984

**Antigen**	**Clone**	**Supplier**	**Order number**
Anti-mouse CD3e BV510	145-2C11	BioLegend	100353
Anti-mouse CD3e BUV395	1451-2C11	BD Biosciences	563565
Anti-mouse CD4 BUV395	RM4-5	BD Biosciences	740208
Anti-mouse CD5 BV510	53-7.3	BioLegend	100627
Anti-mouse CD5 BUV395	53-7.3	BD Biosciences	740206
Anti-mouse CD8a BUV395	53-6.7	BD Biosciences	563786
Anti-mouse NK1.1 BUV395	PK136	BD Biosciences	564144
Anti-mouse CD41 BV786	MWReg30	BD Biosciences	740903
Anti-mouse CD41 PE	eBioMWReg30	ThermoFisher Scientific	12-0411-83
Anti-mouse Ter-119 BV510	TER-119	BD Biosciences	563995
Anti-mouse Ter-119 BV650	TER-119	BD Biosciences	747739
Anti-mouse Ter-119 BUV395	TER-119	BD Biosciences	563827
Anti-mouse Ter-119 APC	TER-119	Proteintech	APC-65149
Anti-mouse CD229 (Ly9) Biotin	Ly9ab3	BioLegend	122903
Anti-mouse/Human/Rat Streptavidin BV421		BioLegend	405226
Anti-mouse CD229 (Ly9) BV421	Ly9.7.144	BD Biosciences	748251
Anti-mouse CD229 (Ly9) BUV805	Ly9.7.144	BD Biosciences	749390
Anti-mouse CD49b PE-Cy7	HMα2	BioLegend	103518
Anti-mouse CD49b AF647	HMα2	BioLegend	103511
Anti-mouse CD49b BV711	HMα2	BD Biosciences	740704
Anti-mouse CD150 (SLAM) BV785	TC15-12F12.2	BioLegend	115937
Anti-mouse CD150 (SLAM) PE-Cy7	TC15-12F12.2	BioLegend	115914
Anti-mouse CD34 FITC	RAM34	ThermoFisher Scientific	11-0341-85
Anti-mouse CD34 AF647	RAM34	BD Biosciences	560230
Anti-mouse CD48 AF700	HM48-1	BioLegend	103426
Anti-mouse CD48 APC	HM48-1	BioLegend	103412
Anti-mouse CD117 (c-kit) APC-eF780	2B8	ThermoFisher Scientific	47-1171-82
Anti-mouse Sca-1 (Ly-6A/E) BV605	D7	BioLegend	108134
Anti-mouse Sca-1 (Ly-6A/E) BV650	D7	BD Biosciences	740450

Antigen	Clone	Supplier	Order number
Anti-mouse CD105 (Endoglin) BV650	MJ7/18	BD Biosciences	740609
Anti-mouse CD135 (Flt-3) PE	A2F10	BioLegend	135306
Anti-mouse CD135 (Flt-3) BV421	A2F10.1	BD Biosciences	562898
Anti-mouse CD127 (IL7-Ra) BV711	A7R34	BioLegend	135035
Anti-mouse CD16/32 AF700	93	ThermoFisher Scientific	56-0161-82
Anti-mouse CD16/32 BUV737	AB93	BD Biosciences	751697
Anti-mouse CD16/32 (Fc-block) Purified	2.4G2	BD Biosciences	553142
